# A Novel Loss-of-Function *MKRN3* Variant in a Chinese Patient With Familial Precocious Puberty: A Case Report and Functional Study

**DOI:** 10.3389/fgene.2021.663746

**Published:** 2021-08-06

**Authors:** Xueling Yin, Junqi Wang, Tianting Han, Zhang Tingting, Yuhong Li, Zhiya Dong, Wei Wang, Chuanyin Li, Wenli Lu

**Affiliations:** ^1^Department of Pediatrics, Ruijin Hospital Affiliated to Shanghai Jiao Tong University, Shanghai, China; ^2^State Key Laboratory of Molecular Biology, Center for Excellence in Molecular Cell Science, Shanghai Institute of Biochemistry and Cell Biology, Chinese Academy of Sciences, Shanghai, China; ^3^Shanghai QingCongquan Training Center for Children With Special Needs, Shanghai, China; ^4^Cancer Center, School of Medicine, Shanghai Tenth People's Hospital, Tongji University, Shanghai, China

**Keywords:** central precious puberty, MKRN3, ubiquitination, GnRH, makorin RING-finger protein 3

## Abstract

**Background:** Central precocious puberty (CPP) is one of the most common and complex problems in clinical pediatric endocrinology practice. Mutation of the *MKRN3* gene can cause familial CPP.

**Methods and Results:** Here we reported a Chinese patient bearing a novel MKRN3 mutation (c.G277A/p.Gly93Ser) and showing the CPP phenotype. Functional studies found that this mutation of MKRN3 attenuated its autoubiquitination, degradation, and inhibition on the transcriptional activity of *GNRH1, KISS1*, and *TAC3* promoters.

**Conclusion:** MKRN3 (Gly93Ser) is a loss-of-function mutation, which attenuates the inhibition on GnRH1-related signaling, suggesting that this mutant can lead to central precocious puberty.

## Introduction

Normal puberty initiation is a process of hypothalamic pituitary gonadal axis activation by pulse secreting of hypothalamic gonadotropin releasing hormone (GnRH) (Yang et al., 2019). The timing of initiation of puberty is determined by the co-regulation of some unknown activation or inhibitory factors. Studies have found that many genes regulate puberty initiation, such as *KISS1, GPR54, LIN28B*, and *MKRN3* (He et al., 2018; Pagani et al., 2020). Mutations or single nucleotide polymorphisms are associated with precocious puberty. Central precocious puberty (CPP) is one of the most common and complex problems in clinical pediatric endocrinology practice. Most CPP patients are sporadic, and almost 30% of them are familial (Li C. Y. et al., 2020). Familial precocious puberty caused by *MRKN3* mutations was first reported by Abreu et al. (2013). So far, more and more *MKRN3* mutation-related familial CPP has been reported (Aycan et al., 2018; Lu et al., 2018; Fanis et al., 2019; Filibeli et al., 2019). The *MKRN3* gene is a maternal imprinted gene, located on chromosome 15q11-q13, which contains only one exon, without introns (Li C. Y. et al., 2020). The gene is highly conserved among different species, and encodes E3 ubiquitin ligases, which participate in the process of selective degradation of proteins in organisms (Abreu et al., 2015; Filibeli et al., 2019).

Our previous study demonstrated that genetic ablation of *Mkrn3* did accelerate mouse puberty onset with increased production of hypothalamic GnRH1. MKRN3 interacts with and ubiquitinates MBD3, which epigenetically silences *GNRH1* through disrupting MBD3 binding to the *GNRH1* promoter and recruitment of DNA demethylase TET2 (Li C. Y. et al., 2020). In this study, a novel *MKRN3* variant (c.G277A/p.Gly93Ser) was found in a Chinese patient with familial precocious puberty, and functional tests indicated it as a loss-of-function mutation.

## Materials and Methods

### Editorial Policies and Ethical

A Chinese patient with a novel *MKRN3* gene mutation was recruited. This study was approved by the Institutional Review Board of Ruijin Hospital. Informed consent was obtained from the participant.

### Molecular Investigations

DNA was extracted from peripheral blood leukocytes using a DNA extraction kit (Qiagen, Hilden, Germany). A custom gene panel was designed and used to capture the targeted sequence, covering all exons and flanking sequences (including the 10 bp of introns) of 187 genes which are associated with the growth and development of children. The procedure for preparation of libraries was consistent with standard operating protocols previously described (Dai et al., 2019). The average mean depth for the targeted regions was 370, and 84.4% of the covered exons had ≥10 reads. Available reads data were 35.1M. The candidate mutation was confirmed with Sanger sequencing using the following primers for *MKRN3*:

Forward primers: 5′-AGCAAGGGAGGGTGTGTCTG-3′;

Reverse primers: 5′-GAGCCAATCACAGGCAAGGAAAG-3′.

### Plasmids Construction

The plasmids pCDNA3.0-MKRN3-3xFlag, pRK5-HA-UB, pRL-TK, pGL3-basic, pGL3-miniCMV, and pGL3-GNRH1-p were kindly provided by Professor Ronggui Hu (Chinese Academy of Sciences, Shanghai, China). Mutation of MKRN3 (c.G277A/p.Gly93Ser) was introduced by site-directed mutagenesis as previously reported (Xu et al., 2018). The promoter regions of the *KISS1* and *TAC3* genes were amplified from human HEK293T genomic DNA (gDNA) and inserted into the pGL3-basic plasmid, generating pGL3-KISS1-p and pGL3-TAC3-p plasmids.

### Cell Culture and Transfection

Human HEK293T and mouse GT1-7 cell lines were kindly provided by Professor Ronggui Hu (Chinese Academy of Sciences, Shanghai, China) and cultured in Dulbecco's modified Eagle medium (DMEM, Life Technologies, USA) or DMEM/F12 (1:1) medium supplemented with 10% fetal bovine serum (FBS), 100 U/ml of penicillin, and 100 mg/ml of streptomycin (all from Gibco, Layola, USA) in a 37°C humidified atmosphere of 5% CO_2_. Plasmids were transfected into HEK293T cells using Lipofectamine 2000 (Life Technologies, Carlsbad, USA) according to the manufacturer's instructions.

### Immunoblotting

Immunoblotting was done as previously described (Li C. et al., 2020). Briefly, the lysates of HEK293T cells transfected with plasmids were lysed in RIPA buffer (50 mM of Tris–HCl (PH 7.6), 150 mM of NaCl, 5 mM of EDTA, 0.1% sodium dodecyl sulfate (SDS), and 1% NP-40) supplemented with protease inhibitor cocktails (Roche, Germany). The cleared supernatant lysates were incubated with specific antibodies and protein G agarose beads or incubated with Anti-Flag affinity gels. The immunoprecipitants were denatured at 100°C for 10 min in 2 × SDS-PAGE sampling buffer. The inputs, immunoprecipitants, and other cell lysates were then subjected to SDS-PAGE and transferred to a PVDF membrane (Bio-Rad, USA). The membranes were incubated with the appropriate antibodies against GAPDH (1:5000, 60004-1-Ig, Proteintech, China), Flag (1:1000, 20543-1-AP, Proteintech), or HA (1:2000, 51064-2-AP, Proteintech). Secondary antibodies were labeled with HRP, and the signals were visualized using the Tanon 5200 Imaging System (Tanon, China).

### Luciferase Reporter Assays

HEK293T cells were seeded at 0.5 × 10^5^ cells/well in 24-well plates. After overnight culture, cells were transiently transfected with pGL3-GNRH1-p, pGL3-KISS1-p, or pGL3-TAC3-p together with other vectors (pRL-TK, wild-type MKRN3, or its G73S mutant). A total of 48 h after transfection, the cells were harvested, lysed with 5X passive buffer, and subjected to a Dual-Luciferase Reporter assay according to the manufacturer's instructions (Promega, USA). Data are expressed as mean ± SD and analyzed using one-way ANOVA with Bonferroni *post-hoc* test. ^*^P < 0.05 denotes significant difference and ^**^P < 0.01 denotes very significant difference over three independent experiments.

### Enzyme-Linked Immunosorbent Assay

A total of 48 h after the GT1–7 cells were transfected, the complete medium was replaced with serum-free DMEM for 24 h to synchronize the cell cycles. Then, 24 h after co-incubation, the supernatants were harvested and subjected to GnRH1 (Phoenix pharmaceuticals, RK-040-02) concentration analysis according to the manufacturer's recommendations (Li C. Y. et al., 2020).

### *In silico* Analysis of the Variant

The computational algorithms Polyphen2, SIFT, and Mutation Taster were used to predict the pathogenicity of the missense variant.

### Statistics

Data were analyzed by two tailed unpaired *t*-test or one-way ANOVA with Bonferroni *post-hoc* test using GraphPad Prism 7. ^*^p < 0.05 was considered to be significant, ^**^p < 0.01 was considered to be very significant.

## Results

### Clinical Characteristics and Identification of *MKRN3* Gene Mutation

The proband is a 9-year-old girl and her breasts enlarged at the age of 7.5 with knots and tenderness. Her growth velocity (GV) had no significant acceleration (height 128 cm) and the age of bone was not advanced. Her breasts had enlarged significantly with vaginal discharge in the last 9 months, with a GV increase of 11 cm/year. She had not yet started menstruating. She denied a history of supplements and special drugs. Her height was 143.4 cm (1.15SD) and her weight was 46.8 kg. Her BMI was 22.8kg/m^2^. She was well-balanced and had no facial irregularities. No milk or coffee spots were observed, and no deformity of limbs and spine was found. Anthropometric and laboratory parameters for the proband are shown in Table 1.

**Table 1:** Clinical and laboratory characteristics of the proband with *MKRN3* mutation.

**At onset**	**Age (years)**	**7.5**
**At referral**	**Age (years)**	**9**
	**Weight (kg)**	**46.8**
	**Height (cm)**	**143.4**
	**Bone age (years)**	**9**
	**Tanner stage**	**B4**
	**Pubarche stage**	**PH2**
**Hormonal profile**	**Base LH (IU/l)**	**2.5**
	**Peak LH (IU/l)**	**13**
	**Base FSH (IU/l)**	**6.3**
	**Peak FSH (IU/l)**	**13.1**
	**Estradiol (pg/ml)**	**32**
	**Testosterone (ng/ml)**	**0.14**
**Brain magnetic resonance imaging**		**Normal**
**Pelvic Ultrasound**	**Uterus volume (ml)**	**8.7**
	**Ovarian volume (ml)**	**4.6**
	**Follicles diameter (mm)**	**4–6**

Her father was 169 cm tall and his voice changed at 14 years old. Her mother was 163 cm tall and menarched at 12 years old. The patient's target height was 159.5 cm. Her grandfather was 167 cm tall, and her grandmother, who had an early menarche at 9 years old, was 147 cm tall.

**Figure 1:**
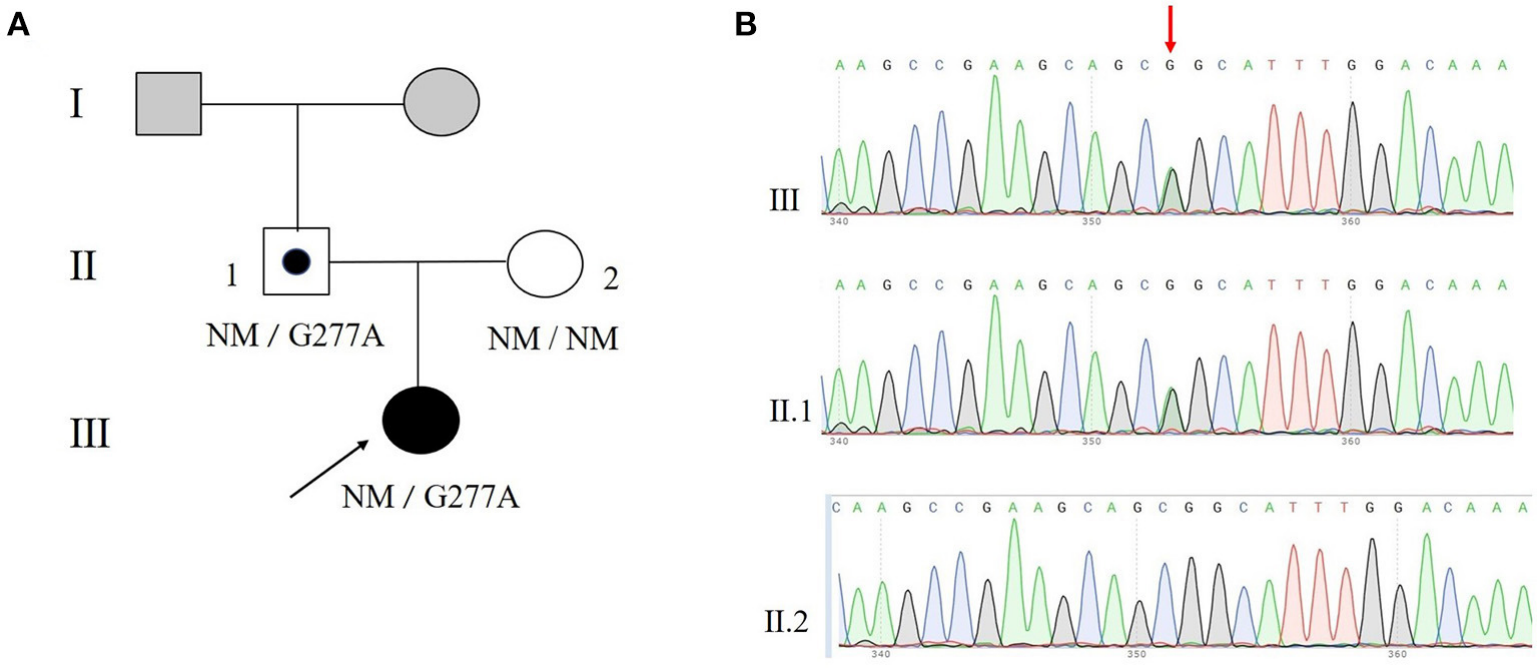
A novel mutation of MKRN3 in a central precocious puberty (CPP) patient. **(A)** Pedigree of a family with a novel c.227G>A mutation in the human *MKRN3* gene. **(B)** Partial sequencing chromatographs of the *MKRN3* gene of the CPP patient and her family members. Squares indicate male family members; circles, female family members; black, individuals with CPP; gray, individuals CPP status unknown; symbol with black dot inside, asymptomatic carriers; NM, no mutation allele. The arrow indicates the proband.

**Figure 2:**
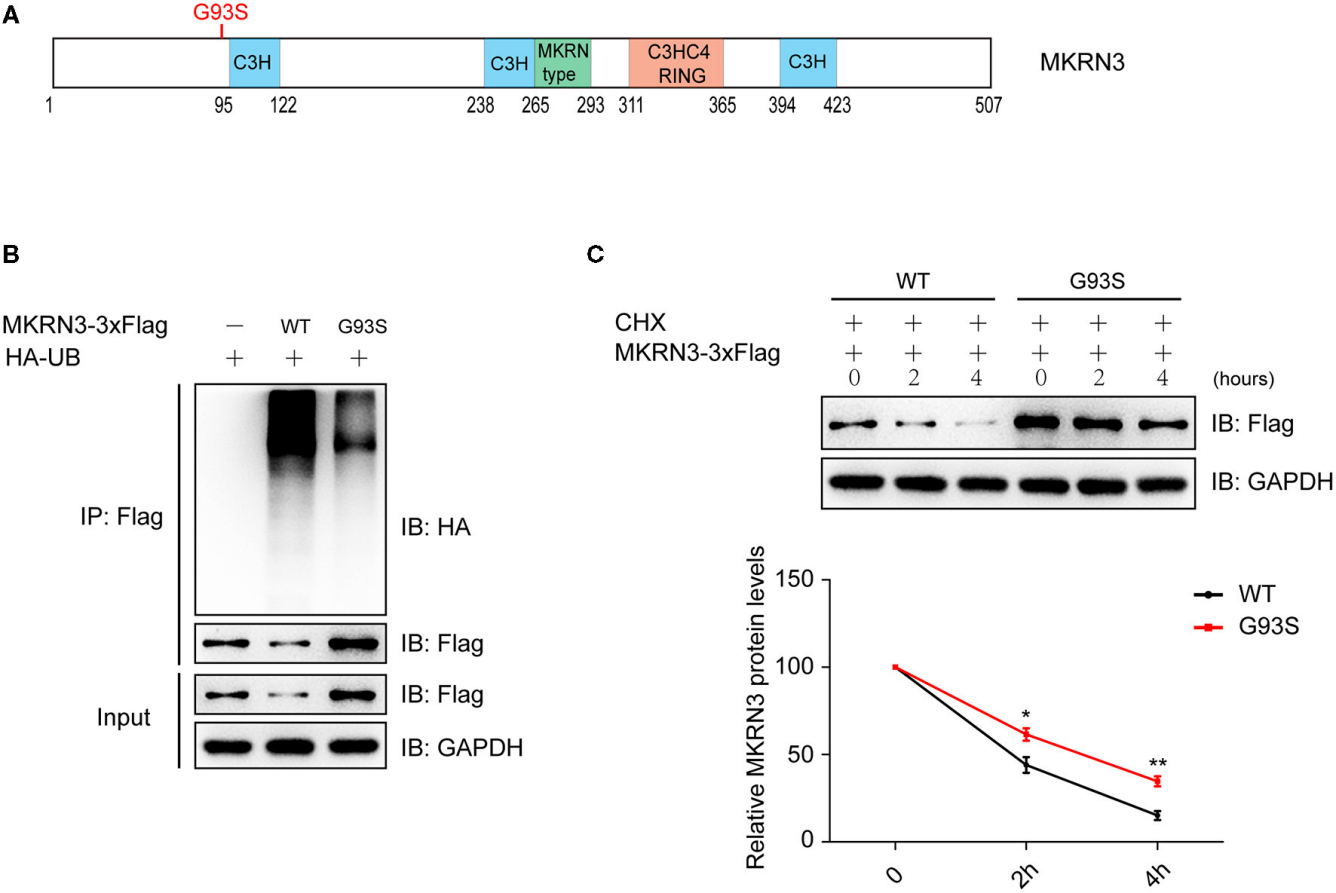
Mutation of MKRN3 attenuated its autoubiquitination and degradation. **(A)** Schematic view of human MKRN3 protein mutation involved in this study. H, histidine; C, cysteine. **(B)** Ubiquitination of the wild-type MKRN3 protein was more significant than that of the disease-associated mutant (G93S) in HEK293T cells. Cells were transformed with plasmids encoding HA-Ub and Flag-tagged wild-type MKRN3, or MKRN3 (G93S) mutant. MKRN3 proteins were immunoprecipitated using anti-Flag beads followed by immunoblotting with anti-HA to detect ubiquitination signals. WT, wild-type. **(C)** The wild-type MKRN3 protein was less stable than the G93S mutant. Flag-tagged wild-type MKRN3 or G93S mutant were expressed in HEK293T cells. Cells were treated with CHX (100 ug/ml) at different time durations (0, 2, or 4 h) before harvest for immunoblotting analysis. Data are presented as mean ± SD, one-way ANOVA was used with the Bonferroni *post-hoc* test over three independent experiments. *P < 0.05, significant difference; **P < 0.01, very significant difference, over three independent experiments.

Genes panel analysis indicated that the proband had a novel *MKRN3* gene mutation (Figure 1A). Her mutation was a point mutation (c.G277A) in exon1 of the *MKRN3* gene which leads to glycine (G) being substituted with serine (S) at condon 93 (p.Gly93Ser) (Figures 1B, 2A). Further Sanger sequencing exhibited that her father had the same mutation, and no *MKRN3* gene mutation was found in her mother (Figure 1B).

### *In silico* Analysis of the Variant

We used several *in silico* computational algorithms (Polyphen2, SIFT, and Mutation Taster) to predict the protein function. PolyPhen-2 classified the variant as “probably damaging” with a score of 1, while SIFT predicted that the mutation was neutral, and Mutation Taster thought it was a polymorphism.

### Mutation of MKRN3 Attenuated Its Autoubiquitination and Degradation

The point mutation (p.G93S) is near the C3H domain of the MKRN3 protein (Figure 2A). As revealed by a previous study, CPP-associated mutations compromise the auto-ubiquitination of MKRN3 (Abreu et al., 2020; Li C. Y. et al., 2020). Our study found that the ubiquitination of wild-type MKRN3 protein was more significant than that of the disease-associated mutant (G93S) in HEK293T cells (Figure 2B), and protein stability detected by immunoblotting analysis indicated that wild-type MKRN3 was less stable than the G93S mutant (Figure 2C).

### Mutation of MKRN3 Attenuated Its Inhibition on GnRH1-Related Signaling

**Figure 3:**
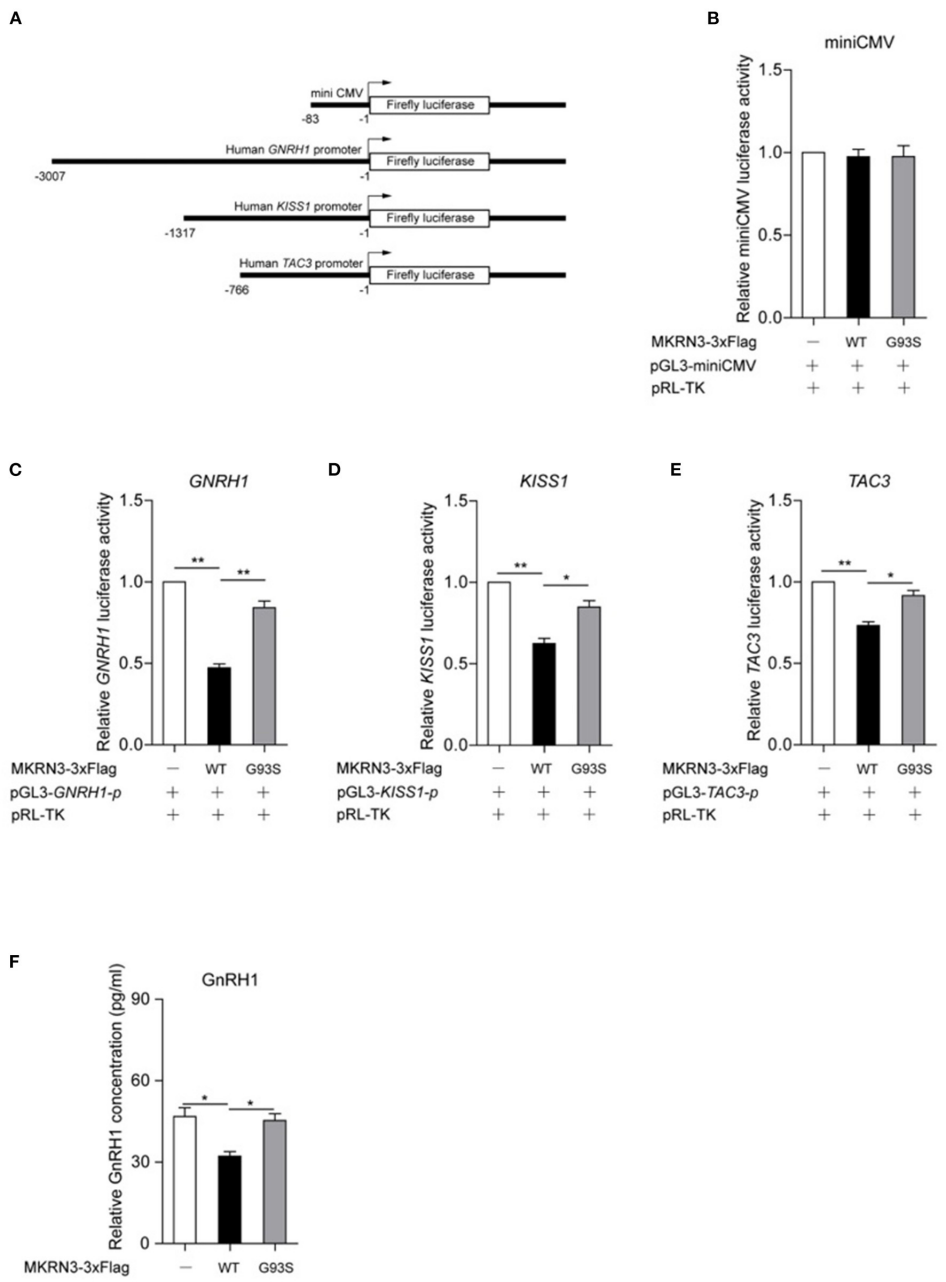
Mutation of MKRN3 attenuated its inhibition on GnRH1-related signaling. **(A)** Schematic diagrams for the construction of luciferase reporter vectors for the human *GNRH1, KISS1*, and *TAC3* gene promoters. The indicated region of promoters was amplified and inserted into the pGL3-basic vector. The miniCMV promoter was inserted and acted as a negative control. **(B–D)** A luciferase reporter assay was used to detect the relative activities of **(B)** miniCMV, **(C)** *GNRH1*, **(D)** *KISS1*, and **(E)** *TAC3* promoters in HEK293T cells. HEK293T cells were transfected with the indicated plasmids and luciferase activities were detected. Data are presented as mean ± SD, one-way ANOVA was used with the Bonferroni *post-hoc* test over three independent experiments. **(F)** Mutation of MKRN3 attenuated its inhibition on GnRH1 levels in GT1-7 cells. GT1-7 cells were transfected with empty vector, wild-type MKRN3, or the G93S mutant. A total of 48 h later, the supernatant was collected and detected by ELISA. Data are presented as mean ± SD, one-way ANOVA was used with the Bonferroni *post-hoc* test over three independent experiments. *P < 0.05, significant difference; **P < 0.01, very significant difference, over three independent experiments.

To study the function of wild-type and mutant MKRN3, luciferase reporter vectors for human *GNRH1, KISS1*, and *TAC3* gene promoters were constructed, and the miniCMV promoter acted as a negative control (Figure 3A). As showed in Figure 3B, MKRN3 showed little activity on the miniCMV promoter. Compared to wild-type MKRN3, the G93S mutant led to weaker suppression of the transcriptional activity of the *GNRH1, KISS1*, and *TAC3* promoters, as revealed in Figures 3C–E. In GT1-7 cells that were derived from mouse hypothalamic GnRH-positive neurons, wild-type MKRN3 significantly repressed the protein level of GnRH1, while the G93S mutant lost this effect (Figure 3F).

## Discussion

Puberty is a transition period from childhood to adulthood with a gradual maturation of the sexual and reproductive systems. Children with central precocious puberty (CPP) show an advanced initiation of hypothalamus-pituitary-gonadal (HPG) axis function, which leads to a rapid development of internal and external reproductive organs and secondary sexual sign before the age of 8 in girls and the age of 9 in boys (Leger, 2002; Kirkgoz et al., 2020). The incidence of CPP is about 1/5,000–10,000, accounting for more than 30% of the total number of pediatric endocrine outpatients (Abreu et al., 2015; Li C. Y. et al., 2020). Children with CPP will suffer from short stature in adulthood as a result of a premature and massive secretion of sex hormones that accelerate bone maturation and premature epiphyseal fusion (Bodicoat et al., 2014). Pre-mature breast development and early onset in girls increases the risk of breast cancer in adulthood and may increase the risk of obesity, diabetes, and cardiovascular disease in the future (Elks et al., 2013; Prentice and Viner, 2013; Bodicoat et al., 2014). The premature development of children's sexual characteristics alongside their immature intelligence and sexual psychology can lead to children's psychological disorders or cause various social problems, so early diagnosis and intervention are needed.

The timing of puberty initiation is thought to be determined by the co-regulation of unknown activation or inhibitory factors. Our recent study demonstrated that MKRN3 acts as an important mammalian puberty initiation regulator (Abreu et al., 2020). A previous study proposed that MKRN3 does not directly alter GNRH1 expression (Yellapragada et al., 2019). However, there are many environmental and metabolic signals postnatally, that may regulate further maturation and function of GnRH neurons. In our study, MKRN3 interacts with and ubiquitinates MBD3, which epigenetically silences *GNRH1* through disrupting MBD3 binding to the *GNRH1* promoter and recruitment of DNA demethylase TET2 (Li C. Y. et al., 2020). As revealed in this study, which was similar to our previous study, MKRN3 represses the transcriptional activity of human *GNRH1* promoter activity, but mutation of MKRN3 attenuates this affect (Li C. et al., 2020). We also found that MKRN3 represses the transcriptional activity of human *KISS1* and *TAC3* promoter activity, which was consistent with the study conducted by Abreu et al. (2020).

The ubiquitin-proteasome system (UPS) mediates the degradation of most cell proteins, and the selection of target proteins is mainly controlled by E3 ligases (Hu and Sun, 2016). CPP-associated mutations compromise the auto-ubiquitination and degradation of MKRN3, including the MKRN3 (G93S) mutant involved in our study (Abreu et al., 2020; Li C. Y. et al., 2020). As revealed in our previous study, MKRN3 mediates the ubiquitination of MBD3 which binds and activates the *GNRH1* promoter, resulting in a low level of GnRH1. Children with loss-of-function *MKRN3* mutations tend to have an advanced initiation of the hypothalamus-pituitary-gonadal (HPG) axis, and a rapid development of internal and external reproductive organs and secondary sexual sign (Abreu et al., 2015). In our study, functional tests indicated that MKRN3 (G93S) is a loss-of-function mutation, which attenuated the inhibition of GnRH1-related signaling, suggesting this mutant can lead to central precocious puberty.

## Data Availability Statement

The raw data supporting the conclusions of this article will be made available by the authors, without undue reservation.

## Ethics Statement

The studies involving human participants were reviewed and approved by Ruijin Hospital Affiliated to Shanghai Jiao Tong University, Shanghai. Written informed consent to participate in this study was provided by the participants' legal guardian/next of kin. Written informed consent was obtained from the individual(s), and minor(s)' legal guardian/next of kin, for the publication of any potentially identifiable images or data included in this article.

## Author Contributions

WL and CL: conceptualization and writing—original draft preparation. XY, JW, and TH: methodology, validation, and formal analysis. WL: investigation. WL, WW, and ZD: resources. XY: data curation. CL, WL, and YL: writing—review and editing. CL: visualization. ZD: supervision. WL: project administration and funding acquisition. All authors have read and agreed to the published version of the manuscript.

## Conflict of Interest

The authors declare that the research was conducted in the absence of any commercial or financial relationships that could be construed as a potential conflict of interest.

## Publisher's Note

All claims expressed in this article are solely those of the authors and do not necessarily represent those of their affiliated organizations, or those of the publisher, the editors and the reviewers. Any product that may be evaluated in this article, or claim that may be made by its manufacturer, is not guaranteed or endorsed by the publisher.
